# Charting an Unknown Protein Universe

**DOI:** 10.1371/journal.pbio.1000206

**Published:** 2009-09-29

**Authors:** Kira Heller

**Affiliations:** Freelance Science Writer, Oakland, California, United States of America

**Figure pbio-1000206-g001:**
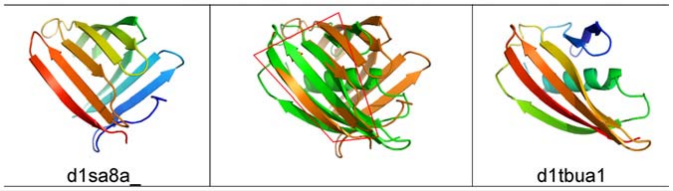
Structural similarities can be seen in subdomains of different folds, as classified by the Structural Classification of Proteins (SCOP) database. In this example, the center column shows the structural superimposition of a pair of partially similar structures, with the first structure on the left and the second structure on the right. An orange contour reveals structurally equivalent residues.

[Fig pbio-1000206-g001]On the surface, the protein universe seems dauntingly vast. Driven by the increasingly rapid accumulation of genomic sequences, the past few decades have yielded sequence data for several million gene products, leaving researchers struggling to keep up. Of the 10,000 protein families listed in the latest release from PFAM (http://pfam.sanger.ac.uk/), an online database that groups proteins based on the similarity of their domains (structural units present in a variety of combinations in different proteins), over 2,100 families are described as domains of unknown function (DUF), because experimental biologists have not yet been able to characterize them.

Historically, structural biologists have focused on exhaustive investigations of the structure and function of individual proteins; although this approach has generated a wealth of information, it is time-consuming and labor-intensive, and it focuses mostly on well-characterized systems, leaving large regions of the protein universe unexplored. The National Institutes of Health Protein Structure Initiative (PSI) has adopted a new approach to structural biology: rather than learning everything there is to know about individual proteins, they are seeking to rapidly characterize the 3-D structures of large numbers of proteins, including many DUF members. As of October 2008, they'd determined the structures of representative proteins from over 250 DUF families.

As reported in this issue of *PLoS Biology*, to learn more about the uncharted regions of the protein universe, Lukasz Jaroszewski and colleagues focused on the sequences and structures of 248 families listed as DUF by PFAM. They found that the DUF families, each of which averaged around 252 protein members, comprised proteins from eukaryotes, bacteria, and viruses; 43% of the DUF families had representatives in more than one of these groups, suggesting that they are evolutionarily conserved and thus likely play important biological roles.

Using sensitive sequence alignment tools, the researchers found that 25% of the protein families designated as DUF could actually be linked to previously characterized protein families; these distant relationships were confirmed by structural comparisons. Another 48% of the DUF families lacked statistically significant sequence similarity to known protein families; however, when Jaroszewski and colleagues combined automated structure comparisons with manual structure analysis, they found that these families can still be classified into known protein folds. And when the researchers decreased the significance threshold for sequence similarity, about half of the families with known protein folds also showed sequence similarity to previously characterized proteins. The remaining 27% of DUF families did indeed contain completely novel folds.

That most of the protein families designated as DUF were in fact structurally similar to known protein families and are likely distant relatives of known proteins was a surprising finding. According to the researchers, this result implies that most of the currently uncharted regions of protein space are actually inhabited by distant relatives of known protein families. Thus, in spite of the vast amounts of sequence data flowing from genome sequencing projects, and even as sequence alignment and structural comparison tools improve, the discovery of truly novel protein families is slowing as the outer limits of the protein universe are explored.

Linking DUF families to previously characterized protein families is an important research tool; researchers can hypothesize biological functions for these unknowns based on these relationships and then experimentally test their assumptions. This approach also tells us something about protein evolution: extreme structural diversification within a relatively small number of protein families provides organisms with the tools to take on new functions and adapt to new environments. And what about those DUF families that are truly novel? They could represent whole new protein galaxies replete with unexpected functions in as-yet undiscovered biological processes, regulatory mechanisms, and structures.

**Jaroszewski L, Li Z, Subramanian SK, Bakolitsa C, Wooley J, et al. (2009) Exploration of Uncharted Regions of the Protein Universe. doi:10.1371/journal.pbio.1000205**

